# Met and unmet care needs of home‐living people with dementia in China: An observational study using the Camberwell Assessment of Need for the Elderly

**DOI:** 10.1111/ggi.14093

**Published:** 2020-11-25

**Authors:** Juxia Zhang, Xiaoqing Xu, LiMei Yang, Jiancheng Wang

**Affiliations:** ^1^ Educational Department Gansu Provincial Hospital Lanzhou China; ^2^ Neurology Department Gansu Provincial Hospital Lanzhou China; ^3^ Out‐Patient Department Gansu Provincial Hospital Lanzhou China; ^4^ Geriatrics Department Gansu Provincial Hospital Lanzhou China

**Keywords:** Camberwell Assessment of Need for the Elderly, dementia, home‐living, met needs, unmet needs

## Abstract

**Aim:**

The goal of the study was to investigate the patterns of needs in older individuals with mild‐to‐moderate dementia living at home using the Camberwell Assessment of Need for the Elderly questionnaire.

**Methods:**

This was a cross‐sectional study. A total of 378 home‐living residents served as the sample. The Camberwell Assessment of Need for the Elderly questionnaire was used to analyze the needs of those receiving adequate interventions (met needs) and those without appropriate supports (unmet needs). Thereafter, the factors that correlated with total needs were determined using demographic characteristics.

**Results:**

Persons with dementia (PWD) had a mean care needs of 18.5 ± 5.4 (range 5–35). Unmet needs were most common in caring for someone (65.1%), looking after the home (63.5%), self‐care (58.7%) and intimate relationships (44.4%) domains. Higher needs were significantly related to living with others than a spouse, longer length of diagnosis, older age and higher cognitive function.

**Conclusion:**

Unmet needs are common in home‐living PWD. Home‐based dementia care can identify and address PWD's unmet needs by focusing on care recipients and caregivers to enable PWD to remain safely at home and improve their quality of life. **Geriatr Gerontol Int 2021; 21: 102–107**.

## Introduction

Dementia is any decline in cognitive ability that has a significant impact on an individual's independence, daily life skills and social interaction.^1^ Due to the lack of effective prevention and treatment measures, approximately 50 million people worldwide are suffering from dementia.^2^ As the population ages, this number is projected to triple by 2050.^3^

Needs can be defined as the capacity to benefit from services. Needs assessment is a process of assessing the needs of individuals, groups or organizations, so as to provide a fundamental base for the needs‐based interventions, and thus affect individuals’ health outcomes.^4^ It is pointed out that only approximately 10% of the symptoms of persons with dementia (PWD) are caused by dementia itself, whereas the remaining 90% are caused by the quality of care they receive.^5^ Dementia care is a long journey challenged by significant PWD and caregivers’ distress, and service demands; however, a review found that there is a gap between the needs and the services actual received.^6^ One study examining the needs of PWD in Australia found that there is lack of support to maintain participation in meaningful activity.^7^ Another study in Baltimore found those with higher cognitive function have greater unmet needs.^8^ Miranda‐Castillo *et al*. highlighted PWD living alone had significantly more unmet needs.^9^ Tapia *et al*. showed that patients’ higher unmet needs were associated with caregivers’ low social support, young age and high anxiety.^10^ Jhang *et al*. found that PWD in Taiwan required more care to prevent traffic accidents and getting lost.^11^ As people with unmet needs reported a lower quality of life, high level of anxiety, increased costs of care,^12^, ^13^ depression^10^ and challenging behaviors,^9^ to provide more appropriate care and support services, it is essential to comprehensively consider the complexity of individual needs, which will assist in targeting services and resources to where PWD most require.

There are several tools available for needs assessment of PWD. The Camberwell Assessment of Need for the Elderly (CANE) is one of them, and was developed by Reynolds *et al*. in 2000, aiming at multidimensional evaluation of the needs of people with mental health problems.^14^ The scale provides an opportunity to analyze the needs from different perspectives that enable health workers to describe the remediable problems in a wide and detailed way. In addition, it has been translated and used in different countries, such as Poland,^15^ Germany,^16^ Portugal,^17^ Korea^18^ and Saudi Arabia,^19^ and is used by researchers worldwide to assess PWD's perception of met and unmet needs in various situations.^6^, ^9^, ^10^, ^12^

By the end of 2019, the Chinese population aged ≥65 years had increased to 176 million, accounting for 12.6% of the total population.^20^ As age increases the risk of dementia, population aging will contribute to a rise in the number of people suffering from any forms of dementia. In China, the number of PWD is estimated to be >9 million, and is expected to exceed 40 million by 2050.^21^ However, dementia care services and related support services are still developing. Influenced by traditional Chinese culture, >90% of PWD live at home and are cared for by their families, whereas only those individuals with mild symptoms and from high‐income households have access to formal care at nursing institutions.^21^ As home care and treatment for disabled and semi‐disabled older persons are not covered by basic health insurance,^22^ such families, especially single‐child families, are under tremendous financial, physical and psychological pressure. In addition, there is a lack of educational programs and support services specially developed for PWD and their caregivers in China. Most family caregivers only provide basic care on eating, dressing and bathing, whereas specialized dementia care, such as cognitive exercise and rehabilitation, is very rare.

The sample hospital is situated in Gansu Province, which is located in the northwest of China. Affected by the regional conditions, the economy, culture and information in Gansu is relatively less developed. ^23^ Currently, there are 349 registered institutions for the aged in Gansu with an average bed occupancy rate of 52.75%.^24^ Of them, 93.98% of institutions provide food services, 89.68% provide cleaning and sanitation services, and only 0.01% provide medical and rehabilitation services.^25^ Up to 2019, there were 4452 employees in these institutions, with just 611 professional and technical staff.^25^ Although the community organizes free medical checkups for older adults aged >65 years every year, the outcomes have not been followed up properly, and risk factors have not been effectively controlled.^21^

As dementia progresses, PWD experience a wide range of needs that can present a challenge for them to live safely at home. As a consequence of the lack of developed health and social services that can meet the needs of PWD, and most of the care relies on informal care, our research on PWD's needs can illustrate how met and unmet needs vary according to the provision of health and social services. In 2006, the Chinese version of CANE was first used by Yeh *et al*. in Taiwan to assess needs the of patients with schizophrenia, and found this instrument is suitable for clinical use in Chinese culture with interrater reliability 0.82–0.98.^26^ However, awareness of CANE's importance among the Chinese population is quite limited. The aims of the present study were to: (i) identify met and unmet needs in a sample of Chinese PWD using CANE; and to (ii) analyze the association between total needs and patients’ sociodemographics, so as to provide more accurate reference for PWD carers and relevant regulatory agencies.

## Methods

### *Participants and setting*

This was a cross‐sectional study. Individuals who attended the memory clinic at Gansu Provincial Hospital in Lanzhou, China, were included. The target group were people with mild‐to‐moderate dementia, living at home and having a carer prepared to participate as well. Exclusion criteria were: (i) severe dementia; (ii) blind/deaf; (iii) severe impairment in communication; (iv) inability to give informed consent; (v) behavior disorders, such as severe aggression, behavior disturbing group work, lack of control or impulsive behavior; (vi) physical disability preventing moving independently; and (vii) somatic ailments requiring pharmacological stabilization; for example, poorly controlled diabetes or arterial hypertension. Consequently, 378 persons were selected. All participants gave their consent after receiving a full explanation of the nature of the study. The study was held from January to April 2019. This study was reviewed and approved by the ethics committee of Gansu Provincial Hospital.

### *Questionnaire*

A questionnaire was used to collect patients’ information. The questionnaire includes two parts: (i) general information, such as age, sex, education level, living status, geographic area, length of diagnose and Mini‐Mental State Examination (MMSE) score; and (ii) CANE scale. The CANE covers 24 areas, which are subdivided into three main domains (psychological, physical and environmental) and two additional domains for caring individuals. For each area, a simple question is posed about a particular need. Responses are rated on a scale where no need is scored 0, met need (problem fully or partially receiving proper intervention) is scored 1 and unmet need (problem left without optimal intervention) is scored 2. Based on the results for each individual, the numbers of met and unmet needs were calculated, as well as the number of all needs as a sum of met and unmet needs. In the present study, the PWD completed the questionnaire. For the analyses, we used the total number of needs (both met and unmet) as an indication of requiring care.

### *Data collection*

Two trained researchers learned the CANE manual before the survey and then carried out the assessment. First of all, a screening of cognitive impairment with MMSE was carried out. MMSE is a standardized, concise and practical cognitive impairment screening tool.^1^ The scale has a total of 30 items, each item has a correct answer score of 1, and a wrong or unknown answer score of 0, with a total score ranging from 0 to 30. Scores of 27–30 indicate normal; scores <27 indicate cognitive dysfunction; scores of 21~26 indicate mild cognitive impairment; scores of 10~20 indicate moderate cognitive impairment; scores of 0~9 indicate severe cognitive impairment. For patients who participate in the survey, the MMSE was scored at least 10 points. Second, the researchers invited the individual to a single room that was next to the clinic. Patients were asked to fill in the questionnaire. If the respondent is unable to answer or cannot answer correctly for some reason (such as illiteracy), the family carers were invited and assisted in filling in the questionnaire. The survey took up to 20 min for each patient. Each time, after the PWD completed the questionnaire, one researcher collected it and placed it in a sealed bag for safekeeping. After all patients completed the questionnaire, the other two researchers entered the data into Excel 2007 (Microsoft Corporation, Redmond, WA, USA).

### *Statistical analysis*

Data was imported from Excel to spss 21 (IBM Corporation, Armonk, NY, USA) for analyses. For all characteristics of studied participants, the mean, SD and median values were calculated, the proportions and means were assessed to determine the number of met and unmet needs. The multiple linear regression analysis was carried out to assess simultaneous independence between variables, specifying the confidence interval with the confidence limit of 95%. One‐way anova was used to compare differences of total care needs between demographic variables (sex, age, education level, living status etc.). *P* < 0.05 was considered to show statistical significance.

## Results

### *General characteristics*

The mean age of the study individuals was 68.3 ± 12.1 years. Among them, 57.1% were men. A total of 40.5% were diagnosed with mild cognitive impairment 3–5 years earlier. The mean activities of daily living of individuals was 61.2 ± 31.5, and MMSE was 23.1 ± 5.8. Most participants (61.1%) were living with others (relatives, adult children), and just 14.9% had paid carers. The detailed characteristics of the study participants are shown in Table 1.

**Table 1:** Characteristics of study participants

Parameter	Characteristic	*n* (%)^a^
Age (years)	<60	81 (21.4)
	60–70	111 (29.4)
	71–80	126 (33.3)
	>80	60 (15.9)
Sex	Female	171 (42.9)
	Male	231 (57.1)
Education	Primary	135 (35.7)
	High school	108 (28.6)
	Degree	39 (10.3)
	Missing data	96 (25.4)
Living status	Alone	0
	With spouse	147 (38.9)
	With others	231 (61.1)
Geographic area	Rural	81 (21.4)
	Urban	297 (78.6)
Length of diagnosis (years)	<1	78 (20.6)
	1–2	129 (34.1)
	3–5	153 (40.5)
	>5	18 (4.8)
MMSE (score)	27–30	102 (27)
	21–26	189 (50)
	10–20	87 (23)
Has a paid carer (yes)		60 (14.9)
Is a carer (yes)		12 (3.0)

Total *n* = 378. MMSE, Mini‐Mental State Examination.

^a^Actual number as a percentage of total study population.

### *Analysis of needs*

The mean needs of all individuals was 18.5 ± 5.4 (range 5–35). Among them, 79.6% were met and 20.3% were unmet.

As shown in Table 2, met needs were noted in most of participants in the following areas: accommodation (97.1%), physical health (81%), medication (77.8%), benefits (61.9%), memory (59.6%) and psychological distress (55.6%). Unmet needs were reported most commonly in the following areas: caring for someone else (65.1%), looking after the home (63.5%), self‐care (58.7%) and intimate relationships (44.4%). Carers reported less needs in information (1.6%) and psychological distress (4.0%).

**Table 2:** Number of participants with reported needs (met and unmet)

Items	Area	No need, *n* (%)	Full/Partially met needs, *n* (%)	Unmet needs, *n* (%)
Environmental needs	Accommodation	0	119 (94.4)	7 (5.6)
	Looking after the home	32 (25.4)	14 (11.1)	80 (63.5)
	Food	106 (84.1)	7 (5.6)	13 (10.3)
	Money/budgeting	49 (38.9)	68 (54)	9 (7.1)
	Benefits	30 (23.8)	78 (61.9)	18 (14.3)
	Caring for someone else	31 (24.6)	13 (10.3)	82 (65.1)
Physical needs	Physical health	2 (1.6)	102 (81)	22 (17.5)
	Medication	6 (4.8)	98 (77.8)	22 (17.5)
	Eyesight/hearing/communication	80 (63.5)	27 (21.4)	19 (15.1)
	Mobility/falls	50 (39.7)	40 (31.7)	36 (28.6)
	Self‐care	34 (27.0)	18 (14.3)	74 (58.7)
	Continence	104 (82.5)	16 (12.7)	6 (4.8)
Psychological needs	Psychological distress	43 (34.1)	70 (55.6)	13 (10.3)
	Memory	30 (23.8)	75 (59.5)	21 (16.7)
	Behavior	46 (36.5)	5 2(41.3)	28 (22.2)
	Alcohol	124 (98.4)	2 (1.6)	0
	Deliberate self‐harm	125 (99.2)	1 (0.8)	0
	Inadvertent self‐harm	120 (95.2)	5 (4.0)	1 (0.8)
	Psychotic symptoms	65 (51.6)	44 (34.9)	17 (13.5)
Social needs	Company	46 (36.5)	40 (31.7)	40 (31.7)
	Intimate relationships	21 (16.7)	49 (38.9)	56 (44.4)
	Daytime activities	35 (27.8)	57 (45.2)	34 (27)
	Information	81 (64.3)	34 (27)	11 (8.7)
	Abuse/neglect	108 (85.7)	11 (8.7)	7 (5.6)
Total		79.6%		20.3%
Mean needs		18.5 ± 5.4 (range 5–35)		
Items for carer	Carer's need for information	106 (84.1)	18 (14.3)	2(1.6)
	Carer's psychological distress	94 (74.6)	27 (22.4)	5(4.0)

Total *n* = 3787.

### *Factors influencing total care needs*

Based on the multivariate linear regression analysis (Table 3), total needs were influenced significantly by the factors as age (B = 1.4, *P* = 0.003), length of diagnosis (B = 1.2, *P* = 0.04), living status (B = 2.9, *P* = 0.002) and MMSE scores (B = ‐0.2, *P* = 0.014).

**Table 3:** Multiple linear regression determinants of total needs

Characteristics	Unstandardized Coefficients		Standardized Coefficients	t	*P*	95% CI	
	B	SE	Beta			Lower	Upper
Sex	1.739	0.622	0.278	2.797	0.051	0.217	3.260
Age (years)	1.452	0.355	0.489	4.093	0.003	0.649	2.254
Length of diagnosis (years)	1.262	0.483	0.274	2.613	0.040	0.080	2.443
Geographic area	0.239	0.105	0.518	2.282	0.042	0.011	0.466
Living status	2.954	0.572	0.579	5.163	0.002	1.554	4.354
Education	−2.210	0.435	−0.479	−5.081	0.002	−3.275	−1.146
MMSE (score)	−0.209	0.061	−0.421	−3.419	0.014	−0.358	−0.059

Total *n* = 378. 95% CI, 95% confidence interval for B; B, Spearman–Brown coefficient; SE, standard error.

One‐way anova analysis (Table 4) showed that needs increased with increasing age (*P* = 0.000, *F* = 8.2), the years of diagnosis rising (*P* = 0.026, *F* = 3.3) and MMSE score increasing (*P* = 0.016, *F* = 3.5). In addition, needs were higher in those staying with others compared with those who stayed with their partner (*P* = 0.044, *F* = 4.1).

**Table 4:** Determinants of total needs (the results of anova analysis)

Characteristics		Total needs (mean ± SD)	*F*	*P*
Sex	Male	18.2 ± 5.7	0.4	0.527
	Female	17.7 ± 5.9		
Age (years)	<60	14.8 ± 1.9	8.2	0.000*
	60–70	15.8 ± 4.8		
	71–80	19.5 ± 5.6		
	>80	21.8 ± 4.8		
Length of diagnosis (years)	<1	15.5 ± 6.3	3.3	0.026*
	1‐2	17.9 ± 4.9		
	>5	22.8 ± 2.5
Living status	With spouse	16.6 ± 6.0	4.1	0.044*
	With others	18.7 ± 5.5		
Geographic area	Rural	18.3 ± 9.6	1.3	0.264
	Urban	16.8 ± 6.3
Education	Primary	17.9 ± 5.7	2.8	0.064
	High school	15.5 ± 5.5		
	Degree	19.1 ± 5.5		
MMSE (score)	27–30	15.6 ± 5.2	3.5	0.016*
	21–26	18.3 ± 5.8		
	10–20	19.5 ± 5.9

Total *n* = 378. ^*^ *P* < 0.05. MMSE, Mini‐Mental State Examination.

## Discussion

There are three types of care modes for older adults in China: home care, community care and institutional care. ^8^ By the end of 2018, China had 155 000 institutions and facilities for the aged, with a total of 7.448 million beds, which means 30.9 beds per 1000 older people.^27^ China's deep‐rooted family traditions, coupled with an underdeveloped dementia service system, have greatly limited the centralized management, treatment, rehabilitation and care of dementia patients. Therefore, the vast majority of dementia patients are still mainly cared for at home, with <5% of those who actually enter the care of a professional institution.^23^ It was also shown in the present study that 85.1% of PWD were looked after by family. This is not only unconducive to the scientific management of PWD, but also leads to exhaustion of family members.^27^ The presented results highlight that based on the opinions of home‐living PWD, dementia‐related needs for care, services and support are often unmet, and similar results were found in community^8^ and long‐term care institutions.^9^, ^15^ This outcome is of interest, as care needs can put PWD at risk of adverse outcomes.^9^, ^10^, ^12^, ^13^ Great understanding of PWD's met and unmet needs can inform the design of care to ensure the services are person‐centered, rather than disease focused.

The present study highlights unmet needs in domains of social (intimate relationships) and environmental areas (caring for someone else, looking after the home, self‐care). Two tasks, caring for someone and looking after the home, had a rate that was >60%. This might be surprising when most studies report conflicting findings regarding these needs.^9^, ^10^, ^15^ The possible explanation is that in China, as the young population moves from the countryside to the city, most children stay in their hometown with grandparents, which is called “left‐behind children” in China. Meanwhile, with the proportion of female employment increasing, most families rely partly or wholly on grandparents to take care of their children and look after the home.^28^ Due to the lack of knowledge about Alzheimer's disease, patients and their families fail to detect, diagnose and treat mild cognitive impairment. As cognitive impairment progresses, it might prevent older people from participating in family care activities (such as cleaning room, cooking etc.), thus impairing their role of caring for the home. This phenomenon might indicate that PWD in China are more concerned about the function of caring for their families than their own psychological and social functions, which is an ideological difference to Western countries. The present study also found that 84.1% of carers have no need for information. This might indicate that the family members still do not realize that they should actively seek ways to meet the needs of older adults. Evidence showed that people who receive more social support from their families, friends and others are more likely to recover from stress,^8^ gain other benefits and obtain higher life satisfaction.^11^ Due to the limited resources available for PWD and their caregivers, we suggest that in future, society should expand the scope of formal care. For the needs that might require less specialist intervention, such as household activities, an effectively support service system should be established for PWD and their caregivers to provide access to resources, information and knowledge.

Self‐care skills are a professional issue, and require professional training to be acquired. In the situation of a large aged population, backward medical technology and insufficient nursing staff, and the lack of self‐care skills is becoming more and more prominent.^8^ This was also shown in the present study and other estimates.^8^, ^28^ Chinese people were found to have a low level of dementia‐related knowledge, especially those aged >60 years and with low education.^29^ In the present study, most PWD had remained in a situation of low education and aged >70 years; meanwhile, most of them suffered from dementia for >2 years, which means they might miss the golden age of treatment and rehabilitation of mild cognitive impairment.^22^ Hence, standardizing the process of early diagnosis, treatment and prevention of cognitive disorders is of great significance in China. At present, there are 128 memory clinics for 1.3 billion people in China, with just 5% of the patients visiting due to Alzheimer's disease.^29^ Hence, it is necessary for China to establish a long‐term care system for PWD. More educational programs should be given to PWD, either through home visits or training, so that they strengthen their sense of responsibility for self‐care, and can take appropriate self‐care measures.

In the present study, compared with people with spouses, the group living with others (adult children caregivers, relatives, friends) had higher needs, similar to the results of other studies.^9^, ^16^ Compared with a spouse, other caregivers are more likely to be juggling multiple roles, which makes it difficult for them to reconcile the roles of care recipients,^27^ they have less contact with the care recipient than spouses would have,^11^ are less involved in daily care and even have less exposure to the care recipient.^15^ Thus leading to PWD being lonely and having significantly higher unmet needs in intimate relationships.^8^, ^9^ On the contrary, spouses have more opportunities to provide company for care recipients and undertake additional caregiving tasks, thus reducing the loneliness of care recipients and having a positive impact on their overall health.^9^ Research found that having a caregiver who was willing to provide services reduced the risk of having unmet needs by 77%.^30^ However, the caregiving tasks make spouses experience more depression and less well‐being.^27^ It has been suggested that caring for PWD is more impactful than other disorders.^11^ A study showed relationship satisfaction is associated with increased suicide risk and depression;^20^ we suggest more frequent assessment and subsequent training programs designed for caregiver samples that are relevant to their situation could be implemented more broadly. In addition, the efforts of academic associations increasingly interested in developing the relevant programs should be encouraged.

Furthermore, higher unmet needs were significantly related to higher cognitive function, which is regarded as another factor associated with an increased number of needs.^8^, ^11^ Cognitive function is associated with activities of daily living and age. Similiar to other studies, the present study also showed that the number of needs was higher in groups with older age.^16^, ^30^ It is pointed out that older adults living at home were two‐ to fivefold more likely to be disadvantaged members, and 93.1% of disabled older people had at least one unmet need.^12^ With worsening disability status, unmet needs increased, as dementia progresses, these older adults with dementia would be the most challenging for caregivers. Specialist nursing support to carers of PWD might have a positive effect on carers’ quality of life, self‐efficacy and subjective well‐being, which enables them to continue providing care on the dementia journey.^13^

We found the length of diagnosis was significantly associated with patient total needs. Patient diagnosed with dementia >5 years had more needs. Patients with long‐term diagnosis had more disease‐related needs in the process of disease development, and these needs had not received timely intervention and attention. China posed “Healthy China 2030” in 2016; furthermore, family doctor contract services were adapted to provide continuous, comprehensive and life‐cycle services to residents.^30^ However, due to an insufficient supply of primary health services and low recognition of contracted services, most residents were unwilling to contract family doctor services.^20^ This might result in untimely medical and non‐pharmacological interventions. As there is no published national dementia policy or strategy in China, the present study shows that recruitment and training of a wider range of health and care professionals and caregivers in a systematic approach to non‐pharmacological interventions could help overcome barriers to the care needs of PWD in resources‐lacking areas.^27^

One of the limitations of the present study was the size of the sample, further evaluation of the CANE based on a larger sample is required to support our findings. Another limitation was that our study only evaluated the needs from the perspective of patients, and did not include the needs of caregivers and staff, further comprehensive analysis is required in the future.

The areas of unmet need identified by CANE can guide health service providers and decision‐makers in addressing important issues in dementia care. By identifying the key factors that influence met and unmet needs of PWD living at home, the present study should provide information on how to optimize the transition between care services actually required by PWD and the services supporting them, especially when there are great differences in care needs among patients at different cognitive levels, age, length of diagnosis and living status, which also affect the depth and breadth of care received, plus family conditions and management strategy should be developed in hierarchical care.

## Disclosure statement

The authors declare no conflict of interest.
